# The Ubiquitin-Like Protein PLIC-1 or Ubiquilin 1 Inhibits TLR3-Trif Signaling

**DOI:** 10.1371/journal.pone.0021153

**Published:** 2011-06-17

**Authors:** Nabanita Biswas, Shufeng Liu, Tapani Ronni, Steven E. Aussenberg, Weiqun Liu, Takashi Fujita, Tianyi Wang

**Affiliations:** 1 Infectious Diseases and Microbiology, University of Pittsburgh, Pittsburgh, Pennsylvania, United States of America; 2 Department of Microbiology, Immunology, and Molecular Genetics, University of California Los Angeles, Los Angeles, California, United States of America; 3 Laboratory of Molecular Genetics, Institute for Virus Research, Kyoto University, Kyoto, Japan; University Hospital Zurich, Switzerland

## Abstract

**Background:**

The innate immune responses to virus infection are initiated by either Toll-like receptors (TLR3/7/8/9) or cytoplasmic double-stranded RNA (dsRNA)-recognizing RNA helicases RIG-I and MDA5. To avoid causing injury to the host, these signaling pathways must be switched off in time by negative regulators.

**Methodology/Principal Findings:**

Through yeast-two hybrid screening, we found that an ubiquitin-like protein named protein linking integrin-associated protein to cytoskeleton 1(PLIC-1 or Ubiquilin 1) interacted with the Toll/interleukin-1 receptor (TIR) domain of TLR4. Interestingly, PLIC-1 had modest effect on TLR4-mediated signaling, but strongly suppressed the transcriptional activation of IFN-β promoter through the TLR3-Trif-dependent pathway. Concomitantly, reduction of endogenous PLIC-1 by short-hairpin interfering RNA (shRNA) enhanced TLR3 activation both in luciferase reporter assays as well as in new castle disease virus (NDV) infected cells. An interaction between PLIC-1 and Trif was confirmed in co-immunoprecipitation (Co-IP) and GST-pull-down assays. Subsequent confocal microscopic analysis revealed that PLIC-1 and Trif colocalized with the autophagosome marker LC3 in punctate subcellular structures. Finally, overexpression of PLIC-1 decreased Trif protein abundance in a Nocodazole-sensitive manner.

**Conclusions:**

Our results suggest that PLIC-1 is a novel inhibitor of the TLR3-Trif antiviral pathway by reducing the abundance of Trif.

## Introduction

Extensive studies in the past decade have now revealed a number of innate immune sensors including the Toll-like receptors, RIG-1, MDA-5, DAI, which recognize molecular patterns present on pathogens and rapidly alert the host to the presence of potentially dangers [1], [2], [3]. Specific to virus detection, cell surface or endosome-associated TLRs recognize viral DNA or RNA and initiate MyD88 or Trif-dependent signaling cascades that activate transcription factors NF-kappaB (NF-κB) and interferon regulatory factors (IRFs) and, ultimately, turn on the transcription of hundreds of cellular genes, of which type I interferons (IFNs) are best known for their antiviral effects. In addition, RNA helicase RIG-I or MDA5 can detect intracellular viral double-stranded RNA and activate NF-κB and IRFs via the adaptor protein MAVS/IPS-1/VISA/Cardif, a CARD domain containing protein that is anchored to the mitochondrial membrane [4], [5], [6], [7]. Interestingly, viruses such as hepatitis C virus (HCV) have evolved mechanism to interfere with the critical innate signaling pathways, allowing escape from the host immune surveillance [5], [8], [9], [10].

Ubiquitin is a 76-amino-acid globular protein that is nearly identical throughout eukaryotes [11]. Ubiquitylation, the process of conjugating ubiquitins to other proteins, plays a pivotal role in the physiological turnover or the degradation of proteins in response to environmental stimuli. Historically, ubiquitylation of a protein is known to target it to 26S proteasome for degradation. It is increasingly realized that ubiquitylation also plays a unique role in regulating multiple cell signaling pathways [12]. For example, the transcription factor NF-κB is normally held in cytoplasm in association with its inhibitor called I-κB. Upon cell stimulation with interleukin -1 (IL-1) or TLR ligands, I-κB is phosphorylated by an I-κB kinase (IKK) complex [12]. The phosphorylation of I-κB triggers its K48-linked polyubiquitylation and its subsequent degradation by the 26S proteasome, which then releases NF-κB for activation [12].

Although multiple studies revealed the involvement of ubiquitylation and de-ubiquitylation in regulating IL-1R/TLR pathways, only one recent report by Chuang and Ulevitch described that the ubiquitin-mediated receptor degradation negatively regulated TLR signaling [13]. In that study, a novel TLR interacting protein termed Triad 3A was demonstrated to be an E3 ligase which interacts with several TLRs. Triad3A induces ubiquitylation of TLR9 and TLR4 but not TLR2, which then targets them for proteasomal degradation. As a result of this, the TLR-mediated activation of NF-κB was suppressed. Most recently, it is shown that Triad3A promoted down-regulation of two TIR domain-containing adapter proteins, TIRAP and Trif, RIP1 which is the essential downstream protein of TNF-α signaling [14], as well as TRAF3 which mediates the RIG-I/MAVS signaling pathway [15]. Thus, ubiquitylation and degradation of innate signaling molecules by Triad3A represents a novel pathway by which the host limits the intensity and duration of innate signaling pathways.

In a yeast two-hybrid screening study, we identified an ubiquitin-like protein PLIC-1 as a potential interacting partner of the TIR domain-containing proteins. Using luciferase reporter assays, we found that PLIC-1potently suppressed the TLR3-Trif-dependent IFN-β promoter activity while exerted minimal effect on TLR4-mediated NF-κB activation or TLR7- or MAVS-dependent IFN-β promoter activity. When endogenous PLIC-1 was reduced by short-hairpin based interfering RNA (shRNA), new castle diseases virus (NDV) became hypersensitive to poly I∶C treatment, reinforcing the idea that PLIC-1 negatively regulates TLR3-pathway. Coimmunoprecipitation (Co-IP) studies revealed that PLIC-1 coprecipitated with Trif, but not TLR3. A GST-pull down further showed that the ubiquitin associated (Uba) domain of PLIC-1 directly pulled down Trif. Subsequent confocal microscopy revealed that PLIC-1 co-localized both with Trif and LC3, an autophagosome marker, in punctate structures within the cytoplasm. Furthermore, overexpression of PLIC-1 decreased the cellular abundance of Trif via a pathway that can be blocked by Nocodazole, but not MG132. Altogether, these data suggest that PLIC-1 is a novel inhibitor of TLR3-Trif signaling pathway by decreasing the abundance of the innate signaling adaptor molecule Trif via the autophagic pathway.

## Results

### Identification of PLIC-1 as a TIR-interacting protein

To screen for novel regulators of TLR-pathway, we conducted a yeast-two hybrid assay using the Clontech Matchmaker III system (Palo Alto, CA) according to the manufacturer's instruction. The intracellular domain (TIR domain) of TLR4 was chosen as the bait. 36 million clones from a human leukocyte cDNA library HL4021B were screened and 6 clones showed strong interaction with bait. After sequencing, all six clones were found to contain partial fragments matching a gene named PLIC-1 in the NCBI database.

PLIC-1 contains an amino terminal ubiquitin-like domain (Ubl) and carboxyl terminal ubiquitin associated domain (Uba) as well as two internal repeats of ∼85 amino acids that contain *Sti* I motifs. PLIC-1 is known to bind to membrane proteins including presenilins and GABA receptors [16], [17]. PLIC-1 was reported to bind ubiquitylated proteins and ubiquitin ligases such as E6AP and βTRCP, and proteasome subunits [18]. These interactions are thought to interfere with normal targeting of proteins for proteasome-dependent-degradation, resulting in enhanced stability potential affecting the signaling pathways in which these proteins are involved.

### PLIC-1 exerted a strong inhibitory effect on Trif-dependent signaling in reporter assays

**Figure 1:**
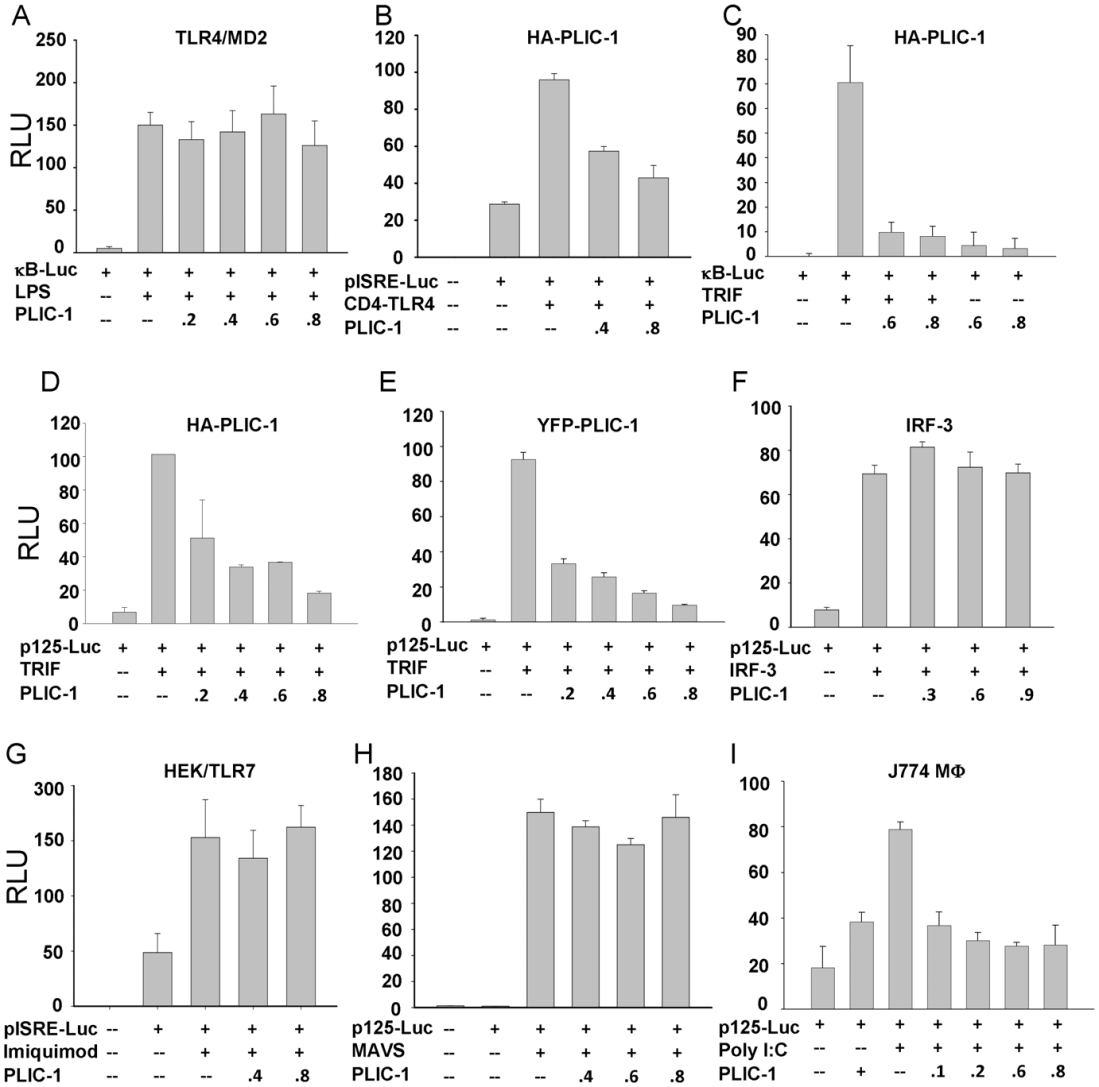
Overexpression of PLIC-1 inhibited TLR3 signaling. **A**. TLR4/MD2 stable HEK cells were transfected with a NF-κB driven luciferase reporter plasmid and increased amount of HA-PLIC-1. 24 hours post-transfection, cells were stimulated with LPS (100 ng/ml) and incubated for additional 24 hours prior to luciferase reporter assay. **B**. 293T cells were transfected with pISRE-Luc (100 ng), CD4-TLR4 (50 ng), and increased amount of HA-PLIC-1 for luciferase reporter assay. **C**. 293T cells were transfected with Trif, κB-Luc, and increased amount of HA-PLIC-1 plasmids. A total of 2 µg DNA was transfected in each well in a 12-well plate. 48 hours following transfection, luciferase activity was determined. Data was normalized against renilla luciferase which was transfected as an internal control. Experiments in **D** were carried out similarly as in **C** except that p125-Luc, plasmid was used. **E**. p125-Luc reporter was transfected along with Trif and YFP-PLIC-1 plasmids. **F**. Experiments were carried out similarly as in **D** except that 0.3 µg IRF3 expression plasmid was added to stimulate p125-luc reporter. **G**. HEK cells stably expressing TLR7 were transfected with indicated constructs and stimulated with TLR7 agonist, imiquimod (10 µg/mL) for 24 hours followed by luciferase reporter assay. **H**. 293T cells were transfected with MAVS and increasing amount of PLIC-1 for reporter assays. **I**. 2.5×10^5^ J774A.1 cells were transfected with indicated plasmids. Poly I∶C(40 µg/ml) was added to cells for additional period of 24 hours prior to measuring luciferase activity. Data were normalized by using renilla luciferase as an internal control. The error bars in above experiments represent the standard deviation accumulated from at least three experiments.

We first investigated the role of PLIC-1 in regulating TLR4 signaling using luciferase reporter assays. To our surprise, overexpression of PLIC-1 had no effect on TLR4-dependent NF-κB activation (Fig. 1A), while modestly suppressed the activation of an ISG promoter driven luciferase reporter by a constitutive TLR4 activator, CD4-TLR4 [19] (Fig. 1B). We then sought to explore its effect on other TLR signalings. To this end, we performed a series of reporter assays and found that PLIC-1 is a potent inhibitor of TLR3-Trif-pathway. First, overexpression of PLIC-1 inhibited the Trif-dependent activation of NF-κB-dependent reporter (Fig. 1C) as well as p125-Luc whose transcription is under the control of IFN-β promoter [20] (Fig. 1D), but had negligible effect on the basal transcription of the reporter gene (data not shown). To rule out the possibility that this inhibition was associated with a specific tagged PLIC-1, YFP- or flag-tagged PLIC-1 constructs were included in the transfection and similar dose-dependent inhibition was observed (Fig. 1E and data not shown). Of note, expression of PLIC-1 in 293T cells did not inhibit the activation of p125-Luc by overexpressing IRF-3, suggesting that PLIC-1 exerts its inhibitory effect at a step that is upstream of IRF-3 (Fig. 1F). To investigate whether PLIC-1 exerts effects on other innate immune signaling, similar studies were repeated on HEK-TLR7 stable cell line and cells transfected with MAVS. It was observed that PLIC-1 failed to have any effect in either case (Fig. 1G&H). Thus, the inhibitory effect of PLIC-1 appeared to be limited to Trif signaling. Because Trif is absolutely required in TLR3 pathway whose activation increases transcription of IFN-β gene [21], we assessed the effect of PLIC-1 on TLR3 activation. In mouse macrophage cells J774A.1 that were stimulated with poly I∶C (TLR3 ligand), the activation of IFN-β-driven luciferase decreased as the amount of PLIC-1 increased (Fig. 1I). Together, these data suggest that PLIC-1 negatively modulates the TLR3-Trif pathway.

### Reduction of endogenous PLIC-1 enhanced TLR3-Trif signaling

**Figure 2:**
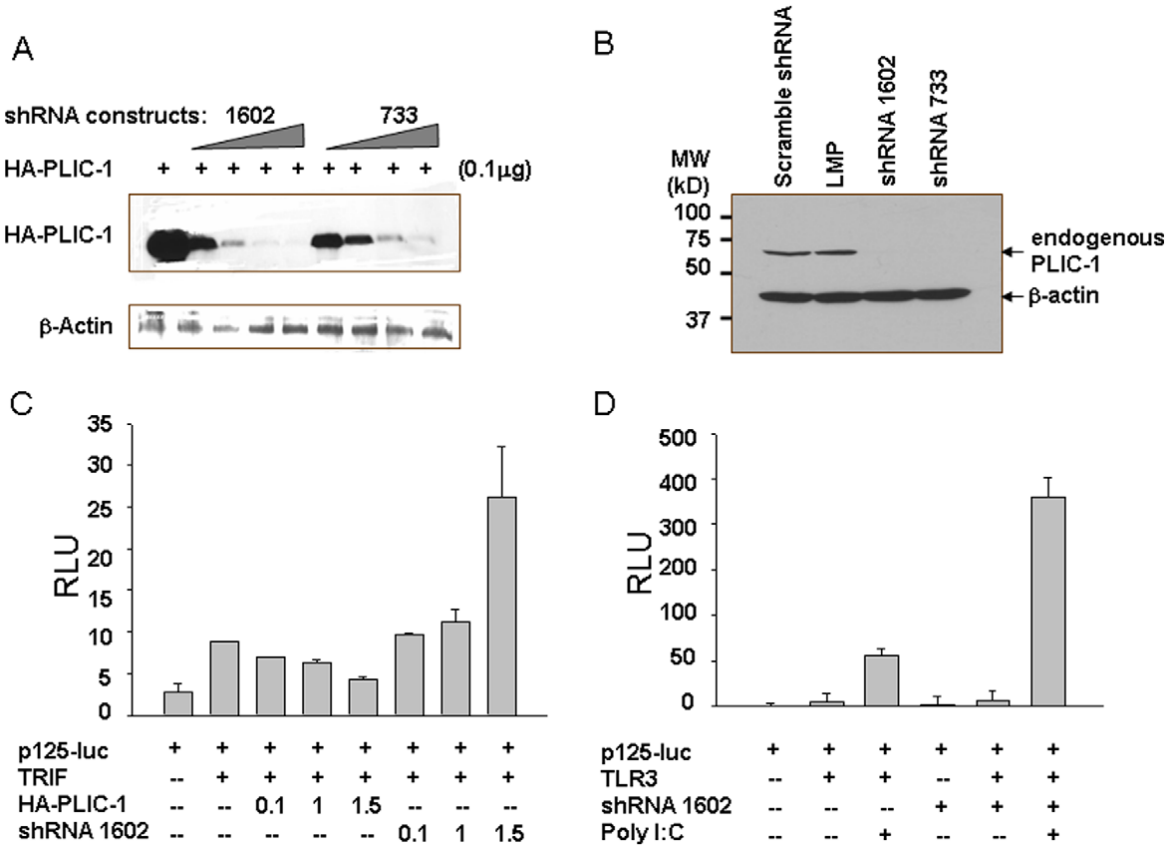
Reduction of PLIC-1 level enhanced TLR3-Trif-mediated signaling. **A**. Knocking down PLIC-1 by shRNA. 0.1 µg of HA-PLIC-1 was transfected with 0.1,1,2,4 µg of indicated shRNA constructs in 293T cells. Western blot was performed to detect HA-PLIC-1. **B**. shRNA 1602, 733, the scramble construct, and LMP (targeting mouse PLIC-1) were expressed in 293T cells. Knockdown of endogenous PLIC-1 was verified by western blotting using an anti-PLIC-1 antibody. **C**. Indicated combinations of constructs were transfected into 293T cells. 48 hours following transfection, luciferase activity was determined and normalized against renilla luciferase. **D**. Reduction of PLIC-1 by shRNA 1602 enhanced the activation of IFN-β promoter activity by poly I∶C stimulation. 293T cells were transfected with indicated plasmids and further stimulated with poly I∶C (40 µg/ml) for 24 hours prior to lysis. Notice that transfection with shRNA construct itself did not activate the IFN-β promoter.

Subsequently, we designed short hairpin interfering RNA (shRNA) knocking down the expression of PLIC-1. The shRNA is expressed from a retroviral vector under the control of human H1 promoter [22]. Two sequences were selected to target three different regions of hPLIC-1. The shRNA harboring a scrambled sequence and the one targeting mouse PLIC-1(LMP) were included as controls for specificity. To monitor the knockdown effect, shRNA vectors were first transfected along with HA-hPLIC-1 construct in 293T cells. Two shRNA constructs targeting to human PLIC-1 specifically suppressed the expression of HA-hPLIC-1 expression in a dose-dependent manner (Fig. 2A). The shRNA construct (#1602) was subsequently employed to reduce the endogenous PLIC-1 (Fig. 2B) and this led to enhanced activation of Trif-dependent IFN-β promoter activity (Fig. 2C). Moreover, transfection with this construct enhanced the activation of the IFN-β-promoter activity (p125-Luc) upon poly I∶C stimulation (Fig. 2D). Taken together, these results reinforced the finding that PLIC-1 inhibits the TLR3/Trif-mediated innate immune signaling activation.

### Reduction of endogenous PLIC-1 rendered NDV hypersensitive to Poly I∶C treatment

**Figure 3:**
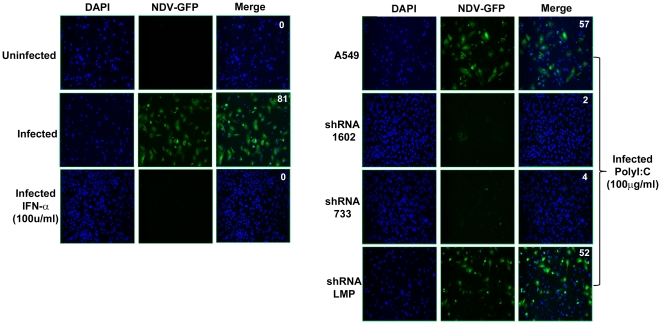
PLIC-1 blocked TLR3-induced production of IFN-α during NDV infection. The parental human lung cancer cell line A549 or the A549 stably expressing shRNA #1602 and 733 (specifically target hPLIC-1) or the negative control shRNA (LMP) were stimulated with poly I∶C (100 µg/ml) for 24 hours and then infected by NDV-GFP (MOI 1). 12 hours post-infection, images were taken to visualize cells that had been productively infected by the green virus. Recombinant IFN-α was added as a positive control to suppress NDV infection. Percentages of positive infection were calculated by averaging the number of green cells over that of blue cells (DAPI staining of nuclei) in 4 fields. The numbers were indicated in the upper right corner of each merged image.

The above experiments were carried out using luciferase reporter assays. In order to validate the observations in an infection system, we generated PLIC-1 stable knockdown cells in the lung epithelial cell line A549 which supports infection of a NDV virus carrying the green fluorescent protein (GFP) in its genome. Because NDV is highly sensitive to type I interferon treatment, such a system allows rapid and sensitive monitoring of bioactivity of type I interferon. Shown in Fig. 3, polyI∶C stimulation of A549 cells modestly reduced the NDV-GFP infection, while the inhibition was significantly enhanced in PLIC-1 knockdown cells. When the bioactivity of type I interferon was compared by calculating the percentage of NDV infected cells, PLIC-1 knockdown A549 cells showed higher level of type I interferon activity. Altogether, these results reinforced that notion that PLIC-1 is an inhibitor of the TLR3 pathway.

### PLIC-1 interacts with Trif

**Figure 4:**
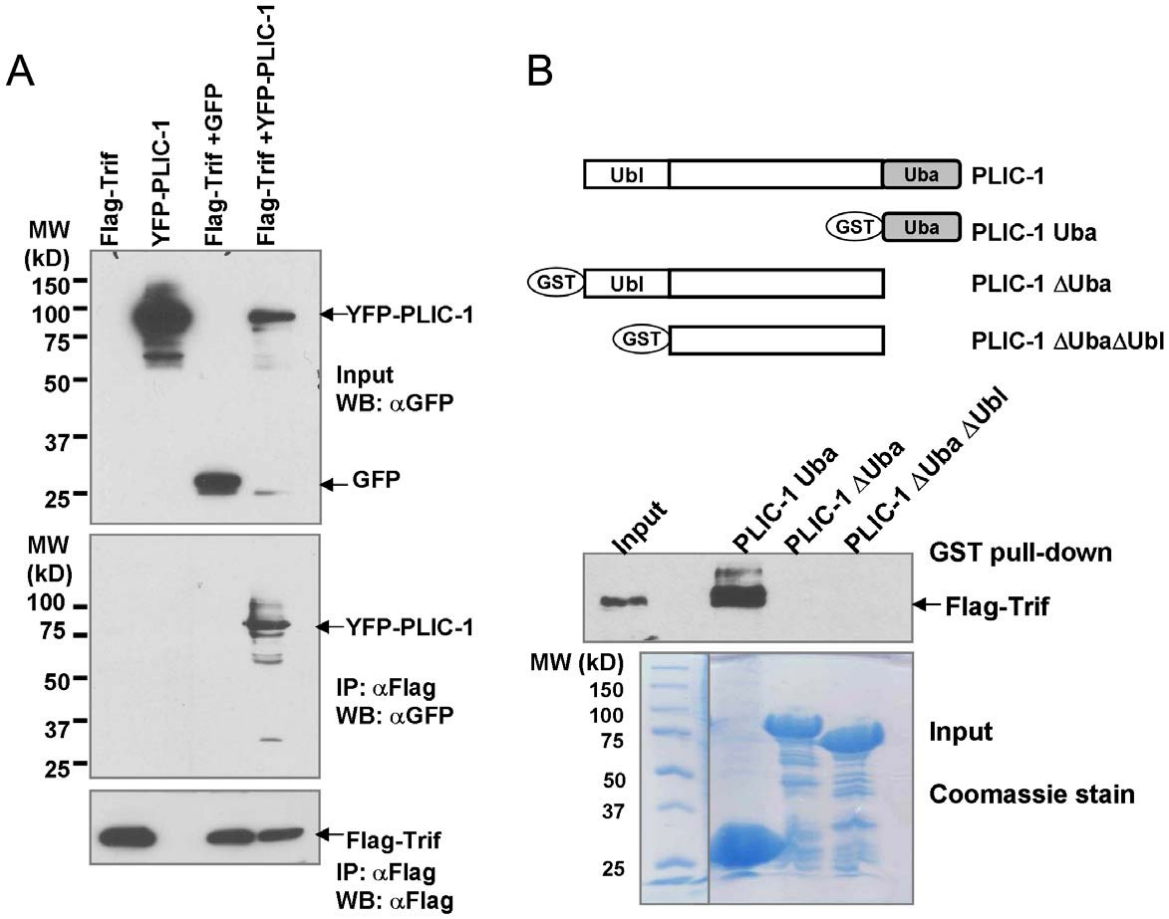
Interaction of PLIC-1 and Trif. **A**. 2 µg YFP-PLIC1or GFP and 2 µg Flag-Trif were cotransfected into 293T cells at 70% confluence in a 60-mm plate. 48 hours post-transfection, cell lysates were prepared and subjected to M2 anti-Flag affinity resin. After extensive wash with PBS, proteins were eluted by boiling the beads in 2× sample buffer and separated on a SDS-PAGE. Western blotting was performed using indicated antibodies. **B**. GST fusion proteins (around 2 µg) were mixed with cell lysates containing Flag-Trif for 2 hours and bound proteins were eluted from the GST column and analyzed for the presence of Flag-Trif. One tenth of the input protein was loaded. A Coomassie stained gel image was included showing the expression of each GST fusion protein.

To explore the interaction between PLIC-1 and Trif, we first conducted Co-IP experiment. Fig. 4A showed that transiently expressed PLIC-1 was able to precipitate co-expressed Trif, but not the negative control protein. Moreover, in a separate experiment, we failed to pull down TLR3 and PLIC-1 together (data not shown). In order to determine which domain of PLIC-1 interacts with Trif, we produced GST fusion proteins containing PLIC-1 Uba domain alone, or PLIC-1 devoid of Uba or both Uba and Ubl domain and conducted a pull-down assay with Flag-Trif. It was found that the Uba domain of PLIC-1 alone is necessary and sufficient to bind Trif (Fig. 4B).

### Co-localization of PLIC-1, LC3, and Trif

**Figure 5:**
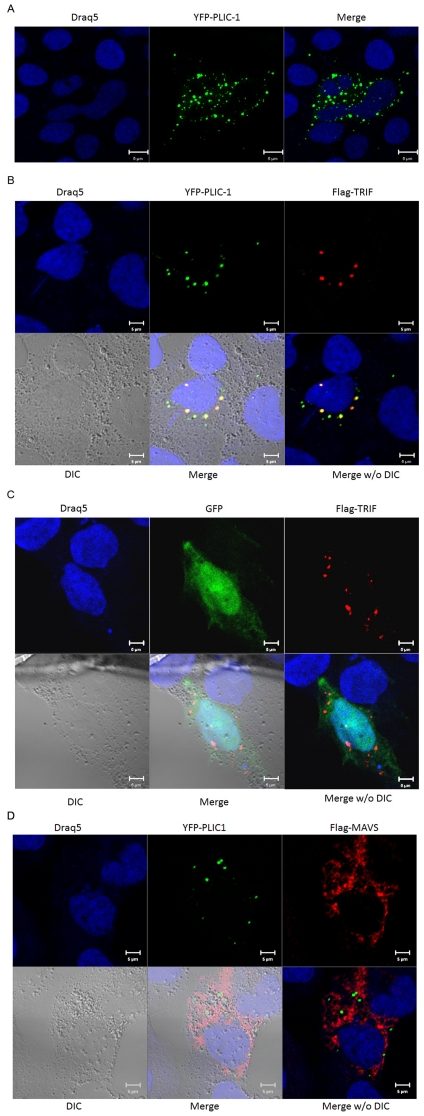
PLIC-1 co-localized with Trif. The human hepatoma cells Huh7.5.1, were seeded on glass cover slip and then transfected with 0.1 µg of YFP-PLIC-1 alone (**A**) or with 0.1 µg Flag-Trif (**B**) in a 24-well plate format. 24 hours post-infection, cells were fixed, permeabilized and stained with anti-Flag M2 antibody. Confocal and differential interference contrast (DIC) images were taken with a Zeiss Meta LSM510 microscope. **C**. 0.1 µg of GFP expression plasmid was transfected with 0.1 µg Flag-Trif for confocal imaging. **D**. 0.1 µg of YFP-PLIC-1 expression plasmid was transfected with 0.1 µg Flag-MAVS into Huh7.5.1 cells for confocal imaging.

Endogenous PLIC-1 was previously documented to concentrate at perinuclear aggresomes [23]. A recent study, however, indicated both PLIC-1 and 2 associate with autophagosomes and depletion of PLICs inhibited autophagosome degradation during nutrient starvation [24]. Trif was found in some speckle-like structures in the cytosol and did not co-localize with TLR3, but transiently associated with TLR3 upon polyI∶C stimulation in the same speckle-like structures [25]. Overexpressed Trif reportedly multimerizes and localizes to punctate cytoplasmic structures referred to as the Trif “signalosome” [23], [24]. To investigate the subcellular distribution pattern of PLIC-1 and Trif, YFP-PLIC-1 and Flag-Trif were transfected into human hepatoma cell line Huh7.5.1 cells and analyzed by confocal microscopy. Huh7.5.1 cells were chosen for this part of the study because they produce very little TLR3 and Trif at endogenous level and have a relatively large volume of cytoplasm [26], which makes them ideal for imaging cytoplasmic proteins. To avoid potential artifact due to overexpression, we intentionally kept the amount of DNA plasmid very low in this study. It was found that YFP-PLIC-1 was concentrated into punctate ring-like structures (Fig. 5A), similar to what has been reported [24], [27]. Immunostaining revealed a rather punctate subcellular distribution pattern for Flag-Trif alone, which is also consistent with previous reports [25], [28]. Of note, we observed a pronounced co-localization of YFP-PLIC-1 and Flag-Trif in punctate cellular structures (Fig. 5B). Of note, GFP itself did not co-localize with Flag-Trif (Fig. 5C). By contrast, there was no co-localization of PLIC-1 and MAVS at all (Fig. 5D). Attempts to image ectopically expressed TLR3 in Huh7.5.1 cells were not successful due to the extremely low level of expression.

**Figure 6:**
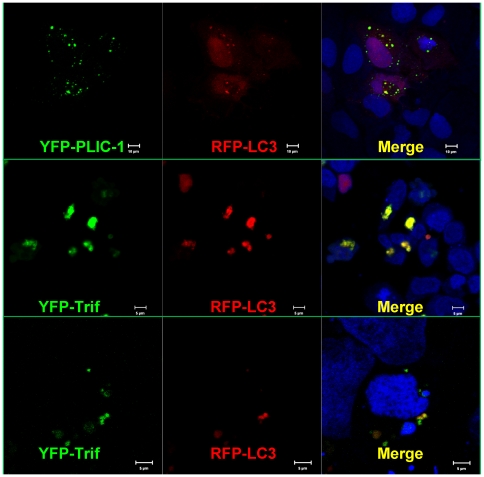
PLIC-1 and Trif co-localized with LC3. The human hepatoma cell line Huh7.5.1 cells were seeded on glass cover slip and then transfected with 0.1 µg of YFP-PLIC-1 and RFP-LC3 (**A**), or 0.1 µg YFP-Trif and RFP-LC3 in HEK (**B**) or Huh7.5.1 (**C**) cells in a 24-well plate format. 24 hours post-infection, cells were fixed and imaged using a Zeiss Meta LSM510 microscope. In all figures, co-localization was indicated as yellow dots in the merged images.

To further explore the nature of the observed vesicular structures, we transfected cells with a RFP tagged LC3 to indicate the formation of autophagosomes. Remarkably, PLIC-1 largely co-localized with LC3 (Fig. 6A). Moreover, when similar experiment was carried out using a YFP-Trif and RFP-LC3, at least a portion of Trif co-localized with LC3 (Fig. 6B&C). These results pointed out that autophagosomes may be where the PLIC-1 and Trif complex locates.

### Downregulation of Trif by PLIC-1

**Figure 7:**
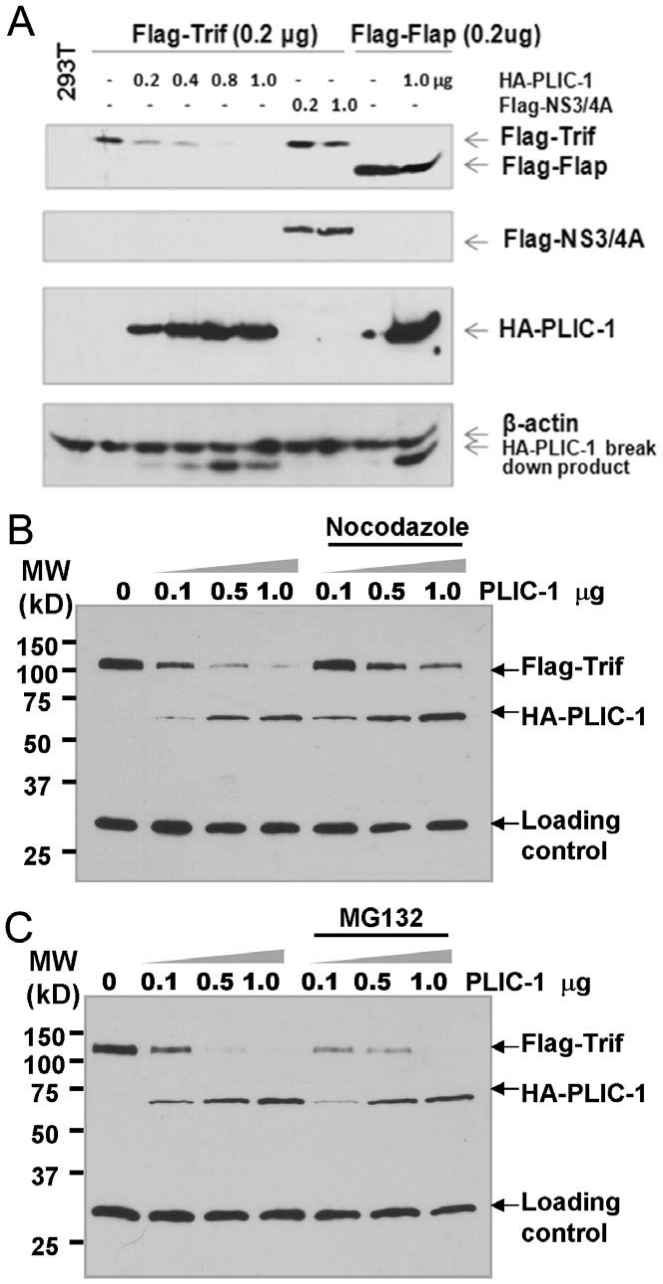
Overexpression of PLIC-1 decreased Trif abundance in a Nocodazole-sensitive manner. **A**. 293T cells were transfected with indicated plasmids. 48 hours post-transfection, lysates were prepared and subjected to western blot analysis. HCV NS3/4A was known to cleave Trif and was included as a positive control [8]. However, it only marginally reduced the level of Trif. PLIC-1 has no effect on the expression of a protein named Flap, which was included as a negative control here. **B**. 293T cells were transfected with 0.2 µg Flag-Trif and indicated amount of HA-PLIC-1 plasmid for 24 hours. Cells were then treated with 30 µM Nocodazole or MG132 (50 µM) for 6 hours prior to cell lysis. Western blotting was performed to quantitate the level of Flag-Trif, HA-PLIC-1. A non-specific band was indicated as the loading control.

In order to gain mechanistic insight into the action of PLIC-1-dependent inhibition, we transfected 293T cells with a plasmid encoding Trif along with specified amounts of PLIC-1 plasmid. After 18 hours, cell lysates were prepared and analyzed by immunoblot of Trif expression. PLIC-1 decreased the abundance of Trif but not an irrelevant protein named Flap (a negative control) in a dose-dependent manner (Fig. 7A). Notably, Flag-Flap was transcribed by a heterologous promoter (CMV promoter) from the same vector in which Trif was cloned. Therefore, PLIC-1 appears to decrease Trif abundance at protein level, presumably via protein degradation.

There are two known pathways for protein degradation: ubiquitin-proteasomal pathway as well as the autophagy-dependent pathways. Since PLIC-1 has been implicated in both pathways, we added the proteasome inhibitor MG132 and the microtubule inhibitor Nocodazle, which is known to block the autophagy-lysosome pathway [29], to the cell cultures. Shown in Figure 7B&C, addition of Nocodazole significantly reversed the PLIC-1-dependent Trif degradation, whereas the effect of MG132 on this process was nearly negligible.

## Discussion

Innate immune signalings mediated by TLRs, RIG-1, and MDA5 are critical in recognizing microbial pathogens and triggering the first line of host defense. Unchecked signaling activation, however, can lead to excessive production of inflammatory mediators that cause tissue damages. Studies in recent years have now revealed a number of negative regulators of innate immune signaling pathways, such as IL-1R-associated kinase M (IRAK-M) [30], CYLD [31], SIGIRR [32], suppression of cytokine signaling 1 (SOCS1) [33], etc. Most recently, Triad3A was demonstrated to act as an E3 ubiquitin-protein ligase and enhance ubiquitylation and proteolytic degradation of several TLRs and RIP1, resulting in the attenuation of TLR signalings [13], [14]. The identification of PLIC-1 now adds a new member to this long list of negative regulators.

PLIC-1 is a type 2 ubiquitin-like (ubl) proteins. In contract to the small-sized type 1 ubl proteins that are covalently linked to target proteins in a fashion similar to ubiquitin, type 2 ubl proteins are not ligated to other proteins and their functions are poorly defined. There are four members of PLIC in mouse and human [18]. Data from functional analyses suggest a role for PLICs in the *in vivo* degradation of several proteins known to be ubiquitylation-dependent substrates of the proteasome [18], [34], [35]. Interestingly, in transfection studies, PLICs either inhibit or promote ubiquitin-dependent proteasome degradation [16], [18], [36]. Moreover, inhibition of G protein signaling by PLIC-1 is independent of proteasome activity [37]. Like ubiquitin, the expression of PLICs is ubiquitous in almost all cell types [17], [31]. Particularly interesting, it is recently shown that PLIC-1 and 2 locate to autophagosomes [23].

Despite that our study initially identified PLIC-1 as a TLR4 interacting partner, we failed to observe any noticeable effect of PLIC-1 on TLR4-dependent NF-κB activation in reporter assays. It must be pointed out, however, that TLR4 signals through MyD88 and Trif sequentially [38] to activate a second-phase signaling. LPS stimulation first triggers TLR4-MyD88-depdent signaling from plasma membrane and activates NF-κB; endocytosis of TLR4 then quenches MyD88-dependent signaling and starts a second phase of TRIF-dependent signaling from endosomes [38]. Because the latter event was more obvious in dendritic cells (DCs) and bone marrow derived macrophages, which were not employed in this study, the observed modest inhibition of PLIC-1 on TLR4-dependent ISG promoter activity could be more pronounced should DCs be examined. Nonetheless, we found that PLIC-1 inhibited the TLR3-Trif-dependent signaling both in reporter assays as well as during NDV infection. TLR3 detects double-stranded RNA (dsRNA), which is a common intermediate during virus replication, and signals exclusively via the adaptor protein Trif. The cell biology of TLR3 and Trif has been very interesting because TLR3 reportedly localizes to endosomes or phagolysosomes [39], [40], [41], [42]. Trif, however, does not co-localize with TLR3 at resting state, but transiently associates with TLR3 upon ligand engagement and initiates the signaling [24]. The exact location of intracellular Trif remains mysterious as immunostaining of Trif did not co-localize with any known early or late endosomal marker [25], [27]. Our finding that Trif co-localized with PLIC-1 and LC3 raises an interesting possibility that Trif may localize to autophagosome. Interestingly, results from the GST pull-down assay demonstrated that the Uba domain of PLIC-1 interacts with Trif, and this is also the domain that is reportedly required for PLIC-1 to target to autophagosomes [24]. Remarkably, ample evidence now highlights a connection between TLR-mediated innate immunity and autophagy. For instance, LPS increased autophagosomes and autophagy-mediated cell death of infected macrophages [43]. In addition, autophagy was shown to be essential for TLR7-mediated recognition of RNA viruses [43]. In future, it will be interesting to determine whether co-localization of Trif and PLIC-1 occurs constitutively or upon TLR3 activation. This likely requires imaging both proteins at endogenous level in cells or near endogenous level in a stable cell line that expresses tagged proteins.

In our study, expression of PLIC-1 diminished the level of Trif when expressed from a transfected plasmid, suggesting PLIC-1 may accelerate the degradation of Trif. Cellular proteins can be degraded via two pathways: the ubiquitin-proteasomal pathway and the autophagic-lysosomal pathway. The ubiquitin-proteasome pathway is fundamental to protein degradation [44]. In brief, ubiquitin is initially activated by the ubiquitin-activating enzyme known as enzyme-1 (E1), and then transferred to the ubiquitin-conjugating enzyme (E2). Together with the ubiquitin-protein ligase (E3), E2 covalently attaches ubiquitins to the target protein at the ε-amino group of a lysine residue. The polyubiquitin chain finally signals 26S proteasome to degrade the ubiquitylated proteins. The ubiquitin-like PLIC-1, however, has no enzymatic activity and is not known to be covalently linked to any protein. The autophagic pathway, on the other hand, offers an alternative mechanism to proteasomes for the degradation of cytoplasmic contents [45], [46]. In this case, portions of cytoplasm and cell organelles are first taken into phagosomes that fuse with lysosomes into phagolysosomes in which proteins will be finally degraded by lysosomal enzymes [47]. Since both PLIC-1 and Trif can localize to autophagosomes and the PLIC-1-dependent effect on Trif is sensitive to Nocodazole treatment, it is tempting to envision that cells may utilize the authophagic pathway that typically degrades cellular components during starvation to degrade Trif signalosome and hence terminate the Trif-dependent innate signaling. Future work is warranted to explore this novel mechanism in detail.

## Materials and Methods

### Cell Lines and reagents

293T (ATCC CRL-11268) cell line and the human hepatoma cell line Huh7.5.1 (gift from Dr. F. Chisari) were cultured in DMEM (Invitrogen Life Technologies) supplemented with 10% FBS (HyClone), 1% penicillin, and 10 µg/ml streptomycin. The mouse macrophage cell line J774 was a generous gift from Dr. David Hackam (University of Pittsburgh School of medicine, PA). J774 cell line was maintained in DMEM supplemented with 5% penicillin and streptomycin, 1% NEAA, and 10% fetal bovine serum (Hyclone). The human lung cancer cell line A549 (ATCC CCL-185) was maintained in F12K media (ATCC 30-2004) supplemented with 10% fetal bovine serum and 5% penicillin and streptomycin. TLR4/MD2 stable cell line was obtained from Dr. D. Golenbock (University of Massachusetts, Worcester). HEK/TLR7 cell line was a generous gift of Dr. S. Saumendra (University of Pittsburgh). Anti-HA, anti-Flag M2, and anti-PLIC-1 (U7383) antibodies were purchased from Sigma.

### DNA constructs

PLIC-1 N terminal and PLIC-2 full length plasmids were obtained from the Howley lab (Boston). The NF-κB-Luc plasmid was obtained from Drs. J. Pomerantz and M. Baldin (Caltech, Pasadena, CA). The p125-luc reporter was provided by Dr. T. Taniguchi (Graduate School of Medicine and Faculty of Medicine, University of Tokyo) and contains the human IFN-β promoter region (−125 to +19) inserted into the luciferase reporter plasmid. The ISRE-Luc plasmid containing 5 copies interferon stimulation response element (ISRE) from ISG54 promoter upstream of the firefly luciferase was a gift of Dr. Kui Li (University of Tennessee Health Science Center). YFP-TLR4, Flag-TLR4, HA-PLIC-1, YFP-PLIC-1, Flag-PLIC-1, Fc-TLR4 and pMIR-DFT-empty plasmid, YFP-Trif, Flag-Trif plasmids were constructed in the lab. RFP-LC3 was a gift of Dr. C. Coyne (University of Pittsburgh). GST fusion constructs were obtained from Dr. E. Brown (University of California, San Francisco). pCL-10A1 which is a retrovirus packaging vector was purchased from IMGENEX. shRNA constructs targeting human PLIC-1 (1602, 733) or mouse PLIC-1 (mLMP), as well as the one with the scrambled sequence, were constructed in the lab (detailed information is available upon request). pFlag-MAVS was kindly provided by Dr. J. Chen (UT Southwestern Medical Center, Dallas, TX).

### Transfection

Transfections of 293T and J774 cells were done using lipofectamine 2000 (Invitrogen) reagent following the manufacturer's instruction. Briefly, plasmids and media (amount depending on the size of the well) were mixed together, and lipofectamine 2000 (2× of the amount of plasmid) was mixed with media in a separate tube. Then media containing plasmids and media containing lipofectamine were then mixed together, allowed to sit for 15–20 minutes at room temperature, and added slowly to the well. After 4 hours the media was replaced with fresh media containing 5% penicillin and streptomycin, 1% NEAA, and 10% fetal bovine serum. For transfection in J774 macrophage cell line, macrophages were transfected according to AFCS (Alliance for Cellular Signaling gateway) protocol of Transfection of Raw Cells. 1×105 cells were plated in each well the day before transfection in 24-well plates. For each transfection, 1 µl Lipofectamine 2000 was added to 25 µl Opti-MEM in a 1.5-ml Eppendorf tube. In another tube the required amount of plasmid was added, mixed, and allowed to sit for 5 min at room temperature. Then, 25 µl DNA mixtures was added to the 25 µl Lipofectamine 2000 mixture and mixed by pipetting. This mixture was incubated for 20 min at room temperature. 200 µl RAWGMI was added to each well and the plasmid/lipofectamine media was then added. After 3 hours the media was removed and replaced with fresh RAWGM1. The composition of RAWGMI media is (for 500 ml) DMEM 435 ml (Final concentration 0.87×), FBS 50 ml (Final concentration 10%), HEPES 10 ml (Final concentration 20 mM), L-Glutamine 5 ml (Final concentration 2 mM).

### Western Blot and Immunoprecipitation

For cell lysate preparation, monolayer cells were lysed with a lysis buffer (50 mM Tris-HCl, pH 7.5, 150 mM NaCl, 0.5% Nonidet P40, 50 mM NaF, 1 mM Na3VO4, 5 mM β-glycerophosphate, 1 mM dithiothreitol, 1 mM phenylmethylsulfonyl fluoride) supplemented with a protease inhibitor mixture (Sigma) on ice. Lysates were cleared by centrifuging at 14,000× g for 20 min. Boiled samples in 2× SDS loading buffer were resolved on a 10–12% SDS-polyacrylamide gel (SDS-PAGE). After electrophoresis, the separated proteins were transferred onto a nitro-cellulose membrane (Bio-Rad). The resulting blots were blocked in 10% milk for 1 h, and then incubated with primary antibody overnight at 4°C. The secondary antibody used in the immunoblot was a 1∶2000 dilution of HRP-linked anti-IgG, followed by detection using the ECL reagents (Amersham). For immunoprecipitation-coupled western blotting, a quantity of 50–100 µg of total soluble extract was incubated with an appropriate amount of beads conjugated with the specified Ab for 2 h at 4°C. Alternatively, the antibody used for IP was added and kept in a rotating condition for 3–4 hrs and then Protein A beads were added and incubated overnight. Next day, after washing, the bound proteins were eluted and then separated on a SDS-PAGE followed by western blotting.

### Reporter assay

For the luciferase reporter assay, 0.25 million 293T cells/well were seeded in 24-well plates. The next day, cells were transfected with lipofectamine 2000 (Invitrogen Life Technologies). A specified amount of DNA was added into each transfection and an empty pcDNA3.1 plasmid was included when necessary (filler DNA) to keep the total amount of DNA same in each transfection. When indicated, cells were treated with various stimuli for specified time periods. Cells were harvested between 24 and 48 h after transfection and luciferase activity was measured using the luciferase assay system (Promega). To control for transfection variations, 0.05 µg Renilla luciferase plasmid whose transcription is under the control of the neutral constitutive HSV-thymidine kinase promoter (pRL-TK) was included in each transfection (internal control). For all experiments, the data presented are the mean ± the standard deviation (SD) of duplicate or triplicate samples and were repeated at least twice.

### NDV-GFP virus production

NDV-GFP virus was a generous gift from Dr. Chris Basler (Mount Sinai School of Medicine, NYC) and was produced in 10-days old embryonated eggs (Charles River Laboratories International, Inc. Wilmington, MA). The inoculated eggs were incubated for two days at 37°C and the allantoic fluid containing virus was harvested, filtered and stored in −80°C until use. The use of this virus has been described elsewhere [48].

### NDV infection assay

0.5 million A549 cells were seeded in 12 well plates twelve hours prior to stimulation. Cells were stimulated in the presence of poly I∶C (100 µg/ml) for 24 hours. Cells were washed with PBS and 20 µl (MOI 1) of NDV-GFP virus was added in 180 µl of PBS (with Ca and Mg) and left at 37°C for 1 hour. Cells were then washed with PBS and incubated in fresh F12K media followed by fixation and imaging. GFP signal started to appear after 12 hours but peaked at 24 hours post-infection. DAPI was added to stain nuclei.

### Immunofluorescence staining and Confocal Microscopy

Transfected Huh7.5.1 cells were fixed in 2% paraformaldehyde (15 min, RT), permeablized with 0.1% (v/v) Triton X-100 in PBS three times (PBST; 10 min, RT), and blocked in 0.3% (w/v) bovine serum albumin (BSA) in PBS (45 minutes, RT), and subsequently incubated in PBS (1 h, RT) with anti-Flag M2 antibody (1∶500). Samples were washed in PBS and subsequently incubated with Alexa 568 secondary antibodies diluted in PBS (1∶500). Nucleus was stained with Draq5 (Biostatus, United Kingdom). Samples were mounted on a slide using a homemade Gelvatol mounting medium. Images were captured on a Carl Zeiss Meta LSM 510 confocal microscope (60× Plan-Neo/1.3 NA Oil) and edited by Photoshop (Adobe).
